# Fire Resistant Adhesive from Chitosan

**DOI:** 10.1021/acs.biomac.4c01467

**Published:** 2025-01-06

**Authors:** Dallin L. Smith, Danixa Rodriguez-Melendez, Maya D. Montemayor, Miguel O. Convento, Jaime C. Grunlan

**Affiliations:** † Department of Chemistry, Texas A&M University, College Station, Texas 77843, United States; ‡ Department of Mechanical Engineering, Texas A&M University, College Station, Texas 77843, United States; § Department of Materials Science and Engineering, Texas A&M University, College Station, Texas 77843, United States

## Abstract

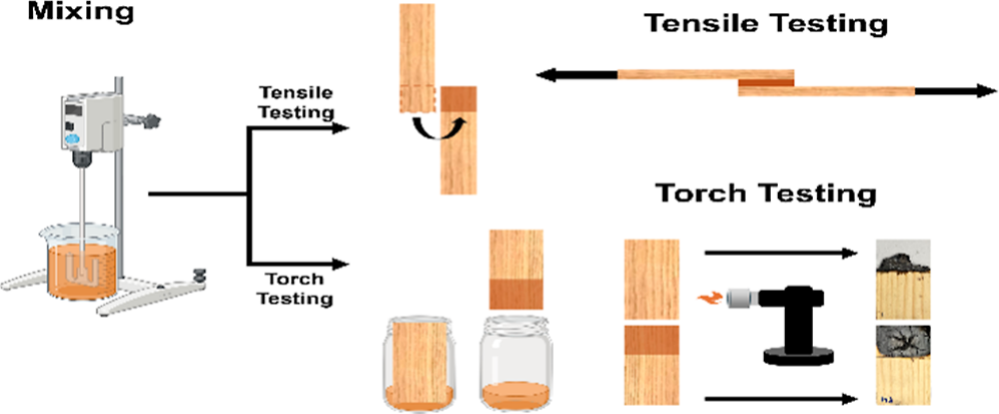

Chitosan is one of the most abundant biopolymers on earth. It is used as a nontoxic alternative in a wide range of medicines, packaging, adhesives, and flame retardants. Chitosan is poorly soluble in neutral or alkaline solutions, but it dissolves in solutions of weak acids, such as acetic acid or citric acid, both of which occur naturally. As a replacement for formaldehyde-containing resins in engineered wood, a chitosan-acid mixture acts as a low-cost, nontoxic adhesive for natural wood that also offers fire protection by forming a char barrier. Pentaerythritol was studied as an additive due to its similarity to glycerol (a common plasticizer for chitosan) and its potential flame retardant benefit. The properties of chitosan adhesives produced with acetic acid and citric acid are compared, and moderate thermal treatment is applied to facilitate covalent bonding (e.g., Maillard reaction) that improves water resistance. Tensile shear strengths of >1 MPa are obtained on lap joints. The unique combination of fire protection and adhesion for wood makes these low cost, biobased systems very appealing.

## Introduction

Engineered wood products (e.g., plywood, oriented strand board, and particleboard) are often assembled with adhesive resins made with formaldehyde.^1^ Despite their durability, water resistance, and low cost, these petroleum-derived thermosets are undesirable due to their harmful emissions.^2^ Recent research describes analogous systems that substitute phenol and formaldehyde with safer or biobased reagents, such as vanillin,^2^ benzaldehyde,^2^ 5-hydroxymethylfurfural,^3^ cardanol,^4^ epoxidized soy oil,^5^ lignin,^1^ and tannins.^6−8^ As safer, more sustainable alternatives to phenol-aldehyde chemistry, some biosourced adhesives are based upon carbohydrates such as starch,^9,10^ glucose,^11−13^ or chitosan.^7,14,15^

Chitosan (CH) is a derivative of chitin, an abundant polysaccharide obtained from crustaceans, fungi, or algae.^16^ Chitosan derivatization involves decalcification, deproteination, decolorization, and deacetylation of chitin.^17^ Unlike chitin, which is insoluble in most solvents, CH dissolves in acidic solutions (pH < 6.0) due to the protonation of the amino group (p*K*_a_ 6.0–6.5).^18,19^ Acetic acid, citric acid, lactic acid, formic acid, phosphoric acid, and hydrochloric acid are common acidic agents for chitosan dissolution.^18,20^ CH is regarded as biodegradable,^21^ nontoxic,^22^ antimicrobial,^23^ and biocompatible^24^ (even edible),^25^ making it a desirable biopolymer for food packaging,^21,25^ adhesives,^7,14,26,27^ drug delivery,^28^ and membranes.^29,30^ It is an attractive biopolymer for many applications because it can pair with other molecules or substrates via covalent, hydrogen-bonding, ionic, and hydrophobic interactions.^20,27^ For example, chitosan was paired with tannic acid to mimic the lysine-dopa chemistry of mussels and outperform commercial adhesives.^14^ Wood is a particularly favorable substrate for chitosan coatings due to its plentiful hydroxyl groups that are available for hydrogen bonding.^26^

A drawback to carbohydrate-based chemistries is their poor water resistance, but this can be improved in CH films via ionic cross-linking or the introduction of a plasticizer.^31^ It is well-established that the properties (e.g., thermal stability, hydrophilicity, and tensile strength) of chitosan films vary with different acids.^19,20,32,33^ The acid’s structure and dissociation strength influence hydrogen and ionic bonding with chitosan, which in turn affect these bulk properties. For example, citric acid is capable of stronger interactions than acetic acid and lowers water vapor transmission rates of chitosan films.^33^ Glycerol is perhaps the most common plasticizer for CH, and it has been shown to act as an internal plasticizer in chitosan by reducing the mobility of acetamide groups and by hydrogen bonding with glucosamine groups.^34,35^ The addition of glycerol also reduces the water absorption of chitosan-citric acid films.^30^ Other polyols (e.g., sorbitol or PEG) have been investigated as plasticizers as well.^34,36^

In addition to adhesives, the chemistry of chitosan also lends itself to flame retardant coatings.^37^ Upon heating, CH forms a stable char, and its hydroxyl and amino groups give rise to inert gases (e.g., H_2_O, N_2_, and NH_3_). In the presence of a dehydrating acid, intumescence can occur.^38,39^ Although chitosan is often utilized in adhesive and flame retardant literature, these two properties are not typically reported for the same system. In this study, CH is dissolved in acetic acid or citric acid with and without pentaerythritol (PER) as an additive. PER was investigated due to its similarity to glycerol and its function as a flame retardant additive. These simple, biobased adhesives exhibit good adhesion to wood and offer protection from fire. Rapid thermal curing improves the water resistance of the adhesive via covalent reactions.^40−42^ The adhesives, with and without thermal treatment and pentaerythritol, are characterized with a number of techniques before and after being applied to wood. Overall, these systems offer a formaldehyde-free, low-cost alternative to other resins in engineered wood products.

## Materials and Methods

### Materials

Citric acid monohydrate (≥98%), pentaerythritol (98%), and glacial acetic acid were purchased from MilliporeSigma (Burlington, MA). Chitosan (95% deacetylated) was purchased from Greentech Biochemicals (Qingdao, China). Solutions were made with 18 MΩ deionized (DI) water. Premium kiln-dried whitewood stud (Home Depot, College Station, TX) was cut into 5.0 × 10.0 × 0.5 cm pieces for torch testing and Radiata Pine (Home Depot, College Station, TX) was cut into 2.5 × 10.0 × 0.1 cm veneers for lap joint adhesion testing.

### Adhesive Preparation

Solutions of 7.5% w/w chitosan were prepared by adding chitosan to a solution of acetic acid (3.57%) or citric acid (12.5%), with or without pentaerythritol (3.15%). For example, to make 100 g of CH–acetic-PER, 7.5 g chitosan was added to a solution of 3.57 g acetic acid, 3.15 g pentaerythritol, and 85.78 g water (92.5 g total). The mixture was stirred (1600 rpm) using an impeller and drill press (Home Depot, College Station, TX) until mostly dissolved. The solution was then placed on mechanical rollers until homogeneous and stored until deaerated before use. The final pH of CH–acetic and CH–citric is 4.5 and 2.5, respectively (with and without PER). Films for spectroscopy, water adsorption, and calorimetry were produced by drying a solution in an aluminum dish at 70 °C overnight. Films that were subsequently placed in a 180 °C oven for 10 min are referred to as “cured”.

### Coating

**Figure 1:**
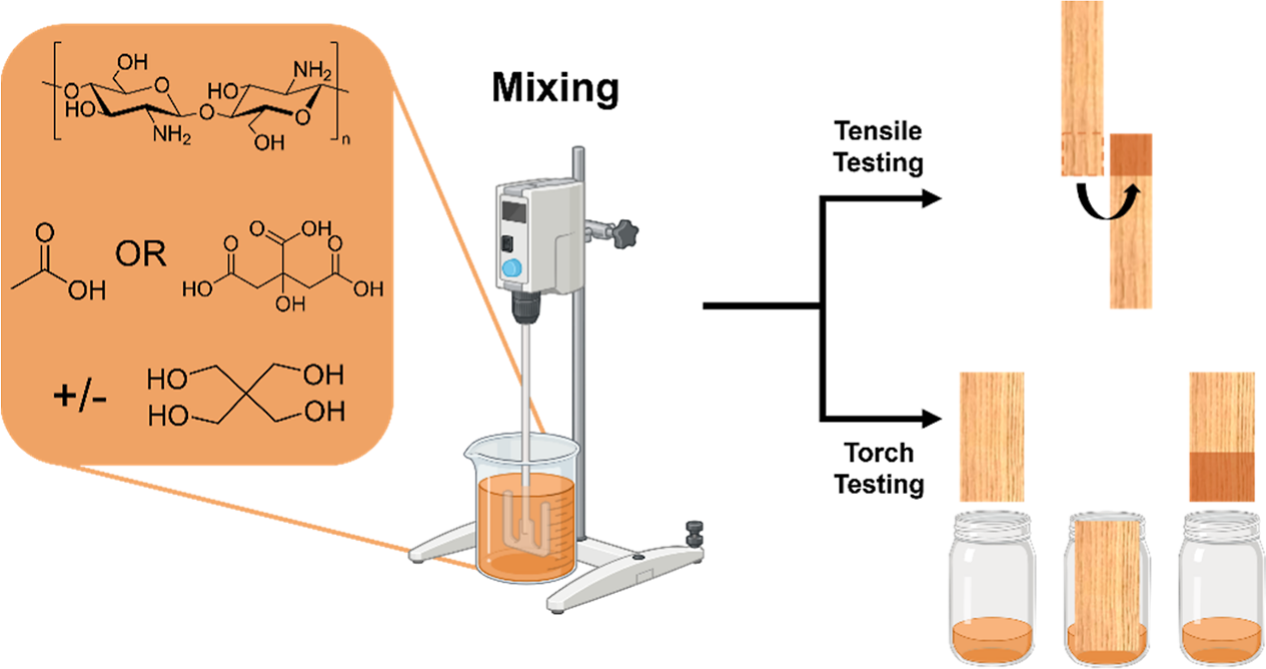
Preparation of samples with flame retardant adhesive.

Wood pieces for torch testing were dipped 1.5″ into adhesive mixture and dried at 70 °C for 1 h. This process was repeated twice more for a total of three cycles. For mechanical testing, lap joints were prepared by spreading 0.15 ± 0.005 g adhesive over 0.0005 m^2^ (300 g/m^2^) and drying in a 70 °C oven. Binder clips were used to keep the lap joints flat during drying. Cured lap joints were subsequently placed in an oven at 180 °C for 10 min. Five samples of each recipe were tested. Adhesive and coated sample preparation is shown schematically in Figure 1.

### Characterization

Flammability was evaluated using a hand-held butane torch (Bernzomatic ST2200T, Worthington Industries, Columbus, OH). The torch was positioned 2 cm away from the sample with a 2 cm inner blue flame and applied for 45 s in the center of the test area. Thermal degradation was studied by thermogravimetric analysis (TGA) on a TGA Q50 (TA Instruments, New Castle, DE). Samples (5–10 mg) were heated at a rate of 10 °C/min under air with 20 mL/min sample flow and 20 mL/min purge flow, after being held at 100 °C for 20 min to remove residual water. Differential scanning calorimetry (DSC) was performed on a Q20 DSC (TA Instruments, New Castle, DE) under 50 mL/min of nitrogen at a ramp rate of 1 °C/min. Fourier transform infrared spectroscopy (FTIR) was performed on a Bruker Alpha Platinum ATR-FTIR spectrometer (Billerica, MA) with a resolution of 2 cm^–1^ and 12 scans averaged. Duplicate ultraviolet–visible (UV–vis) absorption spectra were obtained using a Hitachi U-4100 spectrophotometer (Tokyo, Japan) with tungsten and deuterium lamps at a scan speed of 300 nm/min and slit width of 2 nm. Shear strength of lap joints was evaluated on an Instron 6800 Universal Testing Machine (Norwood, MA) with a 5 kN load cell at 0.25 mm/s and 5″ between clamps. The viscosities of solutions were obtained using a DHR-2 rheometer (TA Instruments, New Castle, DE) at 25 °C with a 40 mm flat plate and a gap distance of 1 mm, in steady-state mode, with the shear rate increased from 0.1 to 1000 1/s. Microscale combustion calorimetry (MCC) was performed according to Method A of ASTM D7309 (Deatak, McHenry, IL). Samples (∼3 mg) were heated at 1 °C/s to 600 °C under a flow rate of 80 mL/min nitrogen, and the thermal degradation products were mixed with a 20 mL/min stream of oxygen before entering a 900 °C combustion furnace.

## Results and Discussion

### Characterization of Adhesives

**Figure 2:**
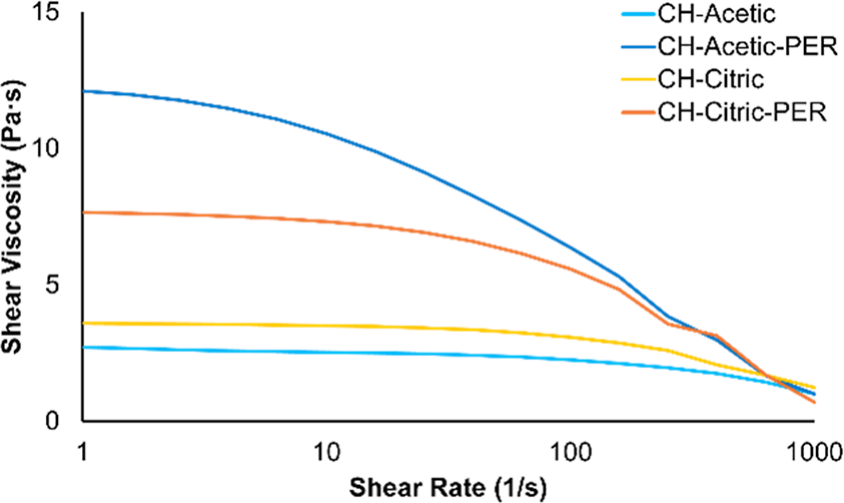
Shear viscosity measurements of each adhesive.

Given sufficient agitation and time, chitosan dissolves in solutions of acetic acid or citric acid at concentrations of at least 7.5% w/w. Conveniently, these solutions can be poured or spread, with viscosities comparable to honey (∼5–10 Pa·s). Solutions made with acetic acid or citric acid have similar viscosities, but acetic acid solutions are more viscous than citric acid solutions in the presence of pentaerythritol (Figure 2). This disparity may arise from citric acid having stronger intermolecular interactions with CH, thus preventing PER from interacting with chitosan to some degree. Concentrated chitosan solutions are known to exhibit shear thinning,^43,44^ but it is more exaggerated for both solutions in the presence of PER. This indicates that PER does interact with chitosan chains (or exacerbates chain entanglement), but to a lesser extent at high shear due to orientation and slipping of chains.^45^ Work by Smith et al. revealed that glycerol disrupts intramolecular interactions in chitosan, yet facilitates intermolecular hydrogen bonding via glycerol-glucosamine assemblies.^35^ Due to its structure, PER is likely to exhibit similar interactions. Although PER was expected to plasticize these solutions, a thickening effect is observed instead, which could be attributed to its relatively high concentration.

Films of each adhesive were created by drying each solution overnight at 70 °C. Based on the remaining mass, about half of the acetic acid is lost to evaporation (Table 1). After 24 h in a humidity chamber (≥99.5% humidity), the acetic acid films adsorb ∼60% of their mass in water, whereas the citric acid films adsorb ∼30%. This disparity could be due to stronger ionic cross-linking present in citric acid.^33^ The addition of PER makes little difference to both CH–acetic and CH–citric. To improve water resistance, films were also heat-treated (“cured”) at 180 °C for 10 min prior to humidity exposure. As a result, water adsorption is significantly reduced to <5% for citric acid films, but remains higher for acetic acid films. Notably, PER increases the water adsorption of cured acetic acid films by ∼50%. This may be connected to the rheological behavior, in which CH–acetic is thickened much more than CH–citric (Figure 2).

**Table 1:** Solids Content and Wet Pickup for Each Chitosan Film after 24 h at ≥99.5% Humidity

recipe	solids (%)	uncured weight gain (%)	cured weight gain (%)
CH–acetic	9.4 ± 0.0	60.7 ± 3.1	35.9 ± 0.9
CH–acetic–PER	12.3 ± 0.1	61.8 ± 3.0	54.7 ± 2.2
CH–citric	19.4 ± 0.1	32.4 ± 1.5	4.6 ± 0.3
CH–citric–PER	22.5 ± 0.0	27.7 ± 1.3	4.9 ± 0.6

FTIR spectroscopy was used to characterize chitosan and dried films of each adhesive before and after curing (Figure S1). Chitosan exhibits absorption at 3300 and 3350 cm^–1^ (−OH and −NH stretches), as well as 1640 cm^–1^ (C=O–NHR stretch), 1585 cm^–1^ (N–H bend), and 1060 cm^–1^ (C–O stretch).^19,30,46^ Once dissolved in acetic acid or citric acid, the N–H bend of chitosan shifts to 1540 or 1520 cm^–1^ (and broadens) due to amine protonation. Acetic acid exhibits a strong carbonyl stretch at 1700 cm^–1^, but it is not observed in CH–acetic films (Figure S1c). Instead, asymmetric and symmetric stretches of the carboxylate appear around 1540 and 1400 cm^–1^, respectively, which overlap with other signals from chitosan.^47,48^ In contrast, a carbonyl stretch from citric acid is still observed at 1700 cm^–1^ in CH–citric films (Figure S1d). The addition of pentaerythritol does not change either spectrum, because it does not contribute new functional groups and hydrogen bonding is already abundant.

After curing, the spectrum of chitosan is unchanged (Figure S1f). In contrast, for CH–acetic and CH–citric, the −OH and −NH stretches consolidate to a single, sharper absorption at 3300 cm^–1^, which could indicate reduced hydrogen bonding of these groups. Additionally, new peaks appear at 1380, 1275, and 1126 cm^–1^ in both CH–acetic and CH–citric, with and without pentaerythritol present. The reason for these specific absorptions is challenging to confidently ascribe, but they may arise from the diverse functional groups in melanoidins produced by the Maillard reaction. The Maillard reaction refers to condensation between nitrogenous compounds and carbonyls (reducing sugars, aldehydes, or ketones), which can be generated during depolymerization of chitosan as a result of heating in acidic media.^40,42,46,49^ Another possibility is amide formation as a result of moderate heating, which has also been reported for chitosan with both acetic acid and citric acid.^30,44^ Both of these pathways would coincide with reduced hydrogen bonding. Curing of each dried adhesive is accompanied by a color change from golden to brown.^44^

UV–vis spectroscopy is used to determine the extent of Maillard reaction. Carbonyls in intermediates absorb 294 nm, and brown melanoidin products absorb 420 nm. The ratio of absorption at 294 nm (*A*_294_) to absorption at 420 nm (*A*_420_) indicates the degree of reaction (i.e., a lower ratio implies more products than intermediates) (Table 2).^40,42,46^ For both CH–acetic and CH–citric, with and without pentaerythritol, *A*_294_/*A*_420_ is lowered by curing, because *A*_420_ increases (Figure S2). The final ratio for all cured films is essentially the same (∼0.95), so it appears that covalent reactions are independent of the solubilizing acid and pentaerythritol. Before curing, pentaerythritol reduces this ratio for CH–acetic films, but slightly increases the ratio for CH–citric, demonstrating again that its effect varies with the acid used to dissolve CH.

**Table 2:** UV–Vis Absorption of Dried Films

recipe	cured	*A*_294_	*A*_420_	*A*_294_/*A*_420_
CH–acetic		1.240	0.892	1.39
CH–acetic	X	1.264	1.315	0.96
CH–acetic–PER		1.167	1.056	1.10
CH–acetic–PER	X	1.213	1.245	0.97
CH–citric		1.106	1.054	1.05
CH–citric	X	1.192	1.278	0.93
CH–citric–PER		1.051	0.914	1.15
CH–citric–PER	X	1.168	1.225	0.95

**Figure 3:**
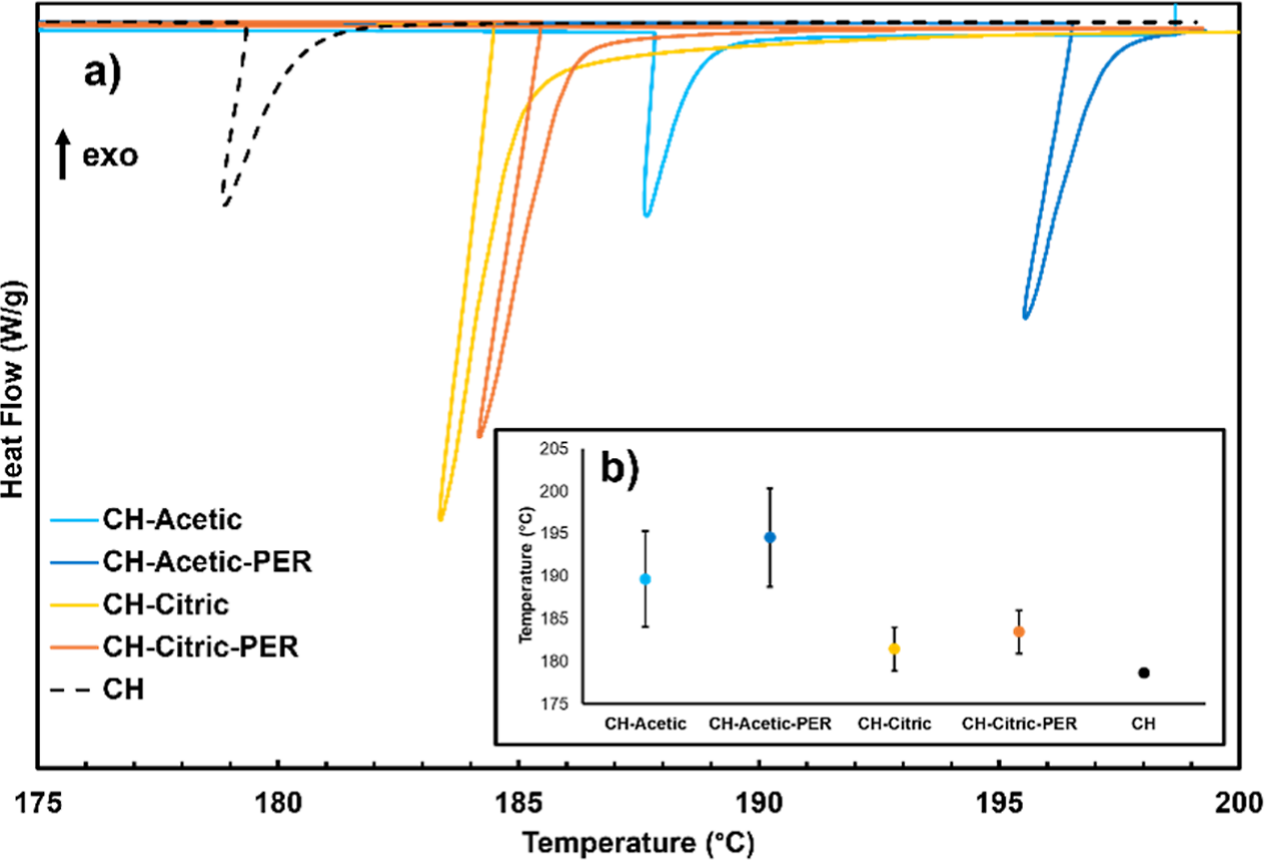
(a) Representative differential thermograms of chitosan (CH) and dried adhesive films. (b) Plot showing average endotherm temperature for each sample, with error bars representing one standard deviation.

An endothermic event is observed in chitosan with DSC at 180 °C, which occurs at higher temperatures for each dried film (Figure 3a). Generally, this event happens at lower temperatures with citric acid than acetic acid, although there is some variation for each recipe. It is unlikely that the endotherm is due to hydrolytic degradation, given that it is observed in the absence of acid, and this effect has been shown to diminish in higher concentrations of CH.^40^ The endotherm may instead arise from loss of strongly bonded water, which has been observed at 168 °C.^50^ Given that CH–acetic films exhibited a higher propensity for water adsorption (Table 1), they likely require higher temperatures to desorb water. Although PER is interacting with the chitosan (evidenced by viscosity measurements), it does not appear to disrupt water removal, as there is no significant delay in this event for either acid (Figure 3b).

### Thermal Stability

**Figure 4:**
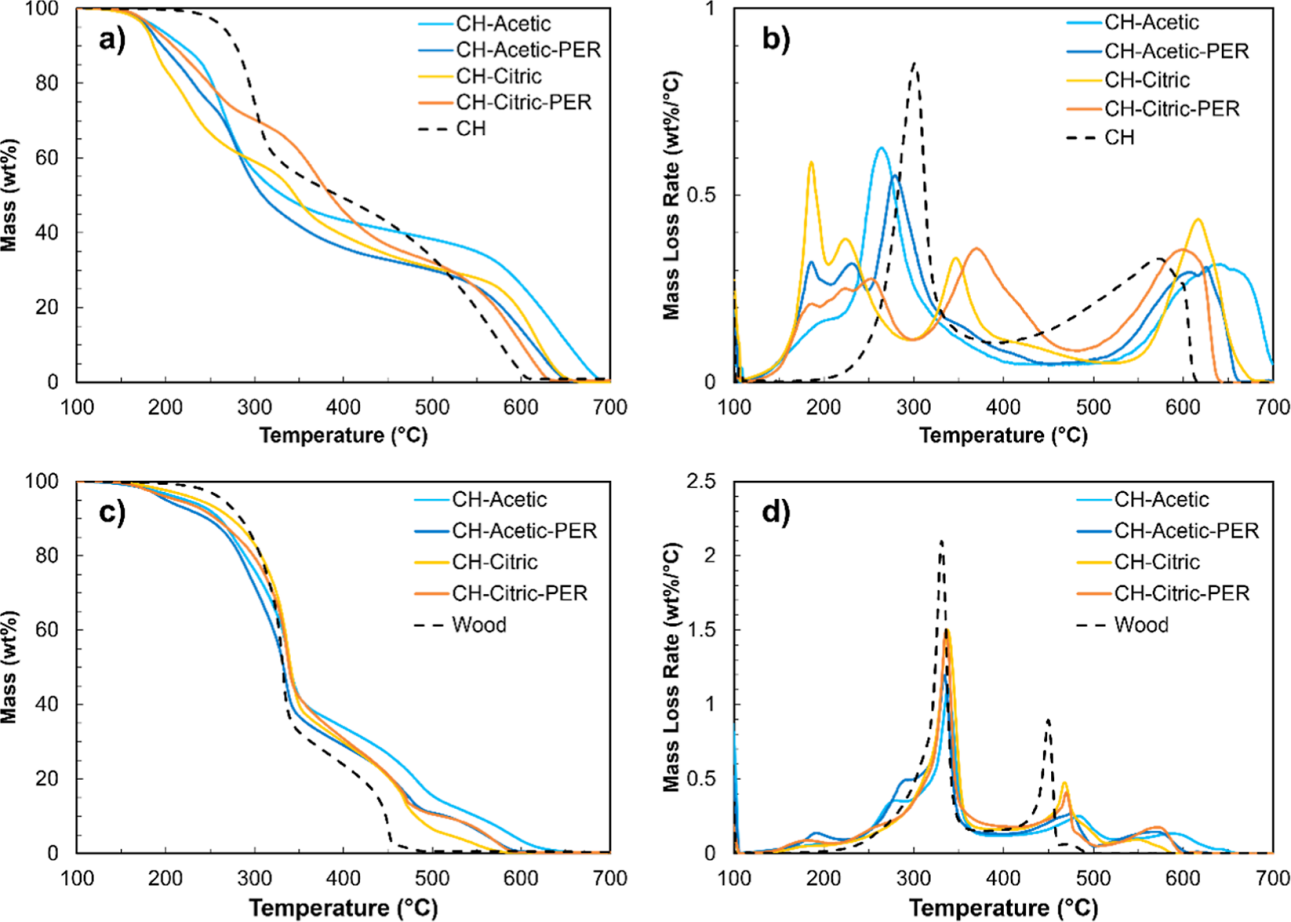
(a) Thermograms and (b) differential thermograms of CH and each adhesive. (c) Thermograms and (d) differential thermograms of wood with each coating.

Thermal stability of the films is characterized by thermogravimetric analysis (TGA). Chitosan exhibits initial degradation under air at 300 °C, due to deacetylation and glycosidic cleavage, and a broad second degradation that peaks at 575 °C, due to thermo-oxidation of cyclic residues (Figure 4a,b).^51^ Chitosan degradation occurs earlier when paired with acetic acid (267–284 °C) and later (348–375 °C) when paired with citric acid, which suggests the reaction pathway with citric acid produces more stable products than acetic acid. Stronger ionic cross-linking in citric acid could also contribute.^20,32^ Early mass loss (<250 °C) can be attributed to evolution of acetic acid, water, ammonia, or acetamide (or citramide).^32,52^ The second degradation event is postponed by 25–75 °C in each system, which indicates a more thermally stable residue compared to chitosan alone. Once again, pentaerythritol has little effect on each system, apart from a slight shift to higher temperatures for the first event and lower temperatures for the second event. Microscale combustion calorimetry confirms the relative stabilities of CH–acetic and CH–citric, as well as the slight delaying effect of PER (Figure S4).

TGA was also performed on wood coated with each recipe to reveal the thermal degradation interactions. Wood also exhibits two-step degradation under air primarily due to hemicellulose and cellulose degradation, respectively, with contributions from lignin (Figure 4c,d).^53^ All coatings delay the second degradation step (450 °C) by ∼20–40°, with no significant influence on the first step (330 °C). Unlike intumescent coatings that include an inorganic acid and accelerate substrate degradation,^53^ these coatings act as a passive barrier to delay thermal degradation by reinforcing (or forming) a char layer.^54,55^ In this case, the acids are merely a vehicle to solubilize and deposit chitosan, which chars,^56,57^ onto the substrate. A final mass loss event is visible beyond 500 °C, which is from the chitosan coatings. Mass loss below 300 °C is also attributed to coating ingredients and byproducts (Figure S3).

### Torch Testing

**Figure 5:**
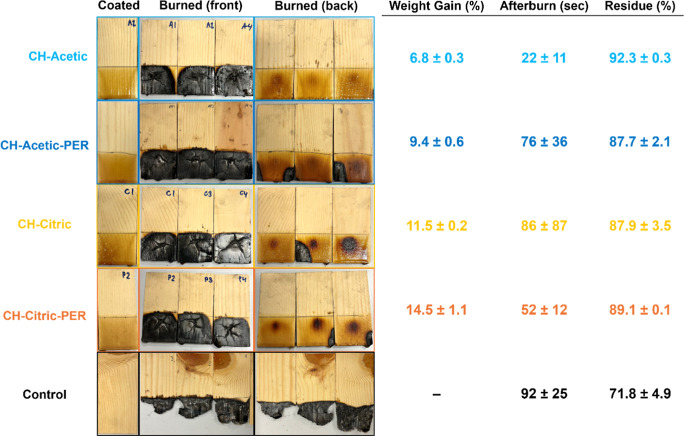
Photos and values from torch testing of wood samples with each coating.

Flame retardants work by inhibiting or removing one or more parts of the fire tetrahedron (fuel, oxygen, heat, and chain reactions) by physical or chemical means in the gas or condensed phases.^58^ Given their chemistry and TGA results, these coatings protect wood from fire by hindering diffusion of oxygen and heat at the surface and releasing inert gases that dilute oxygen and volatiles. Wood samples were exposed to a butane torch flame for 45 s to observe burning behavior. Figure 5 shows photos of samples before and after burning and quantitative results (afterburn and residue). Only the bottom 1.5″ of each 4″ sample was coated and exposed to the flame. Uncoated wood is mostly consumed. Remarkably, CH–acetic is the best performing recipe, despite having the lowest added weight. No char is visible on the back of these samples. The addition of PER worsens the coating, but it slightly improves CH–citric. As a polyol, PER is often included in intumescent systems to provide char via dehydration,^59^ but wood and chitosan can char well on their own, so its benefit is limited in this context. Additional coating mass (above a critical amount) does not offer further benefit.

### Adhesion Testing

The shear strength of lap joints prepared with 300 g/m^2^ of adhesive was evaluated with tensile testing. The strength of the lap joint is calculated as the force at failure divided by the area of overlap. In addition to the interactions among chitosan, acetic acid, citric acid, and pentaerythritol, their adhesion to wood is attributed to the very strong van der Waals and hydrogen bonding to cellulose, hemicellulose, and lignin.^7,26^ Natural wood has a rough, porous surface that also improves permeation of the adhesive.^7^ The favorable compatibility of the adhesive with wood produces adhesive strengths comparable to similar sustainable alternatives for engineered wood products (Table 3). Unlike many of these alternative adhesives, no additional solvents, synthesis, or preparation steps (e.g., hot pressing) are required for the adhesives in this study. Furthermore, none of these other studies report the fire protection of the adhesive.

**Table 3:** Dry Adhesive Strengths of Various Sustainable Adhesives on Wood Substrates

shear strength (MPa)	components	substrate	adhesive Spread	reference
1.7–2.7	chitosan + acetic acid	3-layer ply	400 g/m^2^	Chen et al^44^
1.1	chitosan + starch	3-layer ply	320 g/m^2^	Xi et al^15^
0.5–5.0	chitosan + tannic acid	lap joint	10,000 g/m^2^	Qie et al^14^
2.3	chitosan + tannin	lap joint	200 g/m^2^	Jiang et al^7^
0.9–1.5	citric acid + glucose	3-layer ply	300 g/m^2^	Li et al^11^
30	tannin + glyoxalated lignin polyol	3-layer ply	250 g/m^2^	Faris et al^8^
6.5	epoxidized soy oil + malic acid + tannic acid	lap joint	350 g/m^2^	Westerman et al^5^
1.5–3.0	dialdehyde cellulose + polyamines	3-layer ply	160 g/m^2^	Liu et al^60^
4.2–4.7	epoxidized starch	lap joint	225 g/m^2^	Tratnik et al^10^
1.2–1.7	Oxidized glucose + polyurea	3-layer ply	150 g/m^2^	Zhang et al^12^
**0.76–1.61**	**chitosan + aceticacid or citric acid**	**lap joint**	**300** g/m^**2**^	**this work**

**Figure 6:**
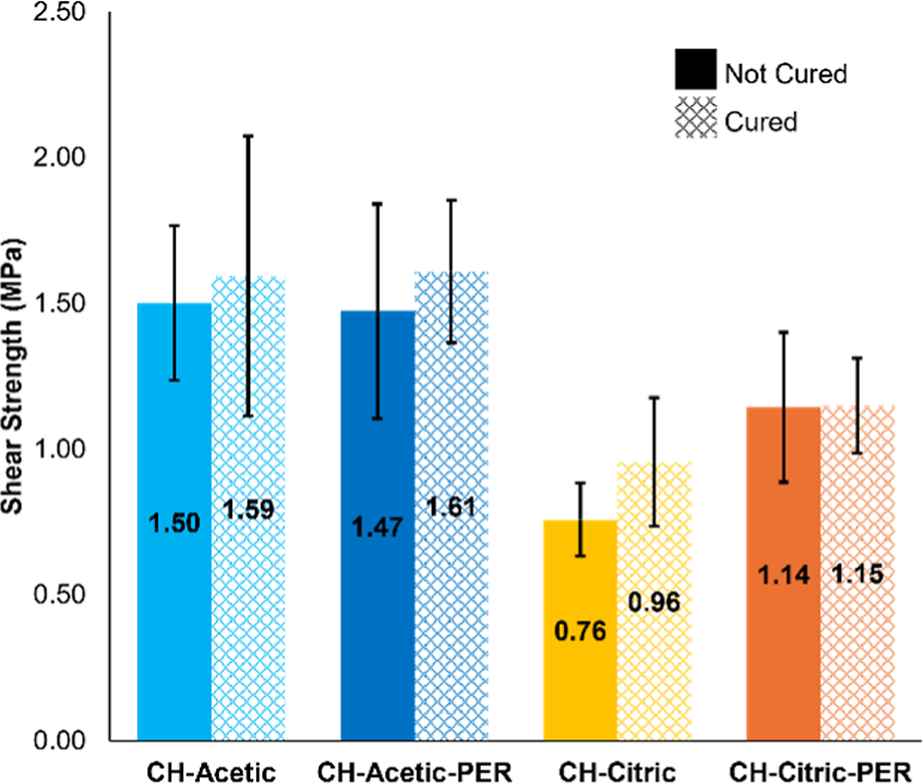
Tensile testing of single lap joints with each adhesive.

As was seen in torch testing, CH–acetic yields the best performance as an adhesive. Generally, the CH–acetic adhesives exhibit a higher shear strength than CH–citric adhesives, despite their lower solids content. Surprisingly, neither pentaerythritol nor curing affects the adhesive strength of either system in a statistically significant way, though it is possible that PER improves CH–citric by a small amount (Figure 6). It is possible that the stronger ionic interactions with citric acid screen or reduce the ability of chitosan to interact with the components of wood, which is crucial for adhesion.

## Conclusions

Biobased adhesives from chitosan are shown to exhibit good adhesion to natural wood and offer fire protection, which is a unique combination of properties. These adhesives require no organic solvents or heating to prepare. Their viscosities are conducive to dipping or spreading and can be modified by the addition of pentaerythritol. One drawback of chitosan-based coatings is their poor water resistance, but this can be improved with a rapid heat treatment, which initiates covalent bonding within the system. Overall, TGA shows a slight improvement in the thermal degradation of coated wood, but torch tests demonstrate the significant protection offered to the bulk material, with only a modest weight gain (∼10%). These low-cost, biobased adhesives present an alternative to formaldehyde-releasing resins for engineered wood products (e.g., plywood). Further work can be done to explore alternative substrates or other additives that benefit the adhesion or the fire protection of these coatings.

## Supporting Information Available

FTIR spectra of reagents and dried adhesives, UV–vis spectra of dried adhesives, differential thermograms of reagents, and MCC curves of dried adhesives (PDF)
