# Supplementary material for: Fire Resistant Adhesive from Chitosan
Source: Biomacromolecules. 2025 Jan 6;26(2):1141–8. doi: 10.1021/acs.biomac.4c01467 (PMC11815856; doi:10.1021/acs.biomac.4c01467)
Supplement: Supplementary file 1 — bm4c01467_si_001.pdf [file bm4c01467_si_001.pdf]

Supporting Information

Fire Resistant Adhesive from Chitosan

Dallin L. Smith,[a] Danixa Rodriguez-Melendez,[a] Maya D. Montemayor,[a] Miguel O. Convento,[b] and Jaime C. Grunlan[a,b,c]*

[a] Department of Chemistry, Texas A&M University, College Station, TX 77843

[b] Department of Mechanical Engineering, Texas A&M University, College Station, TX 77843

[c] Department of Materials Science and Engineering, Texas A&M University, College Station, TX 77843

* Corresponding author

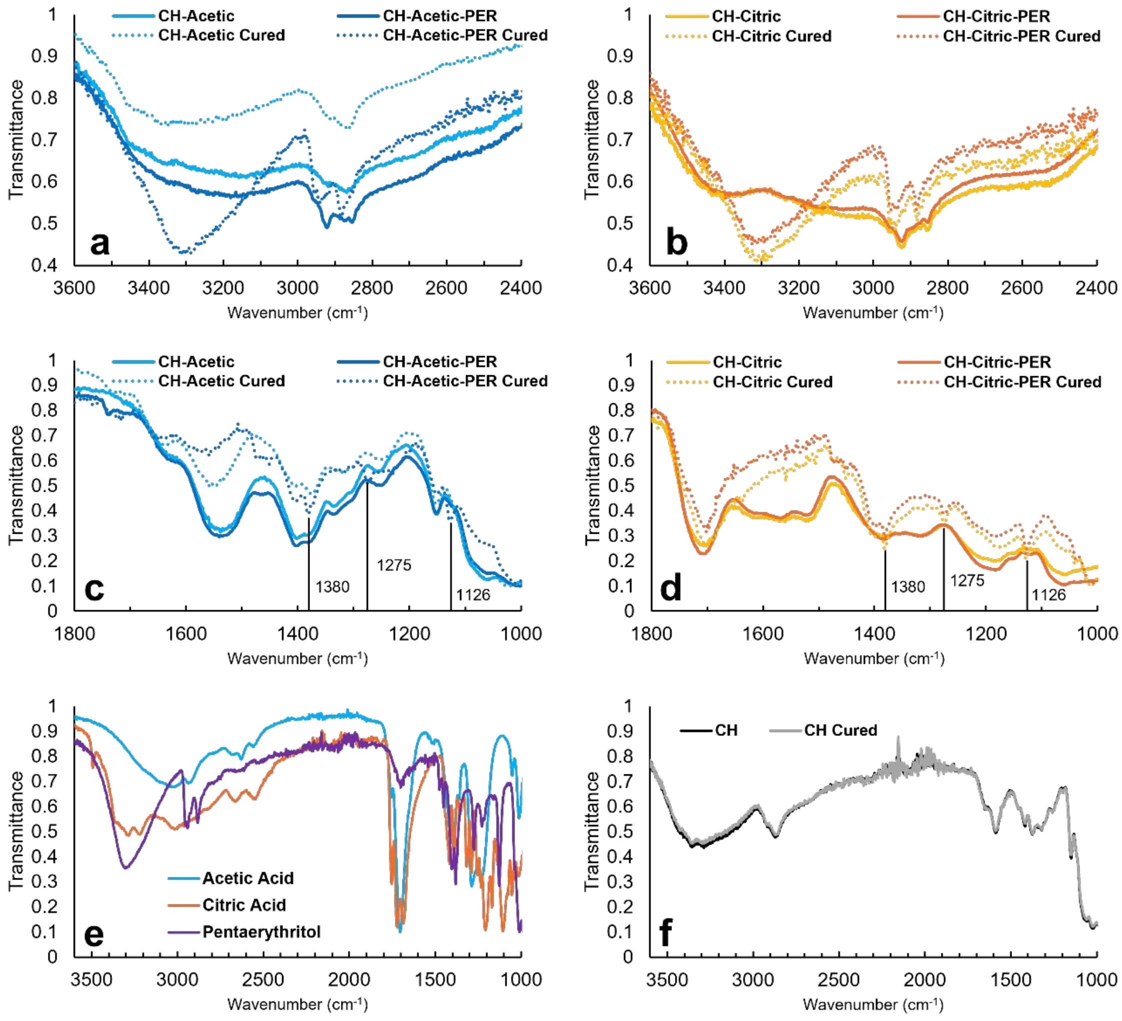

Figure S1. FTIR spectra for dried films of each adhesive and reagents.

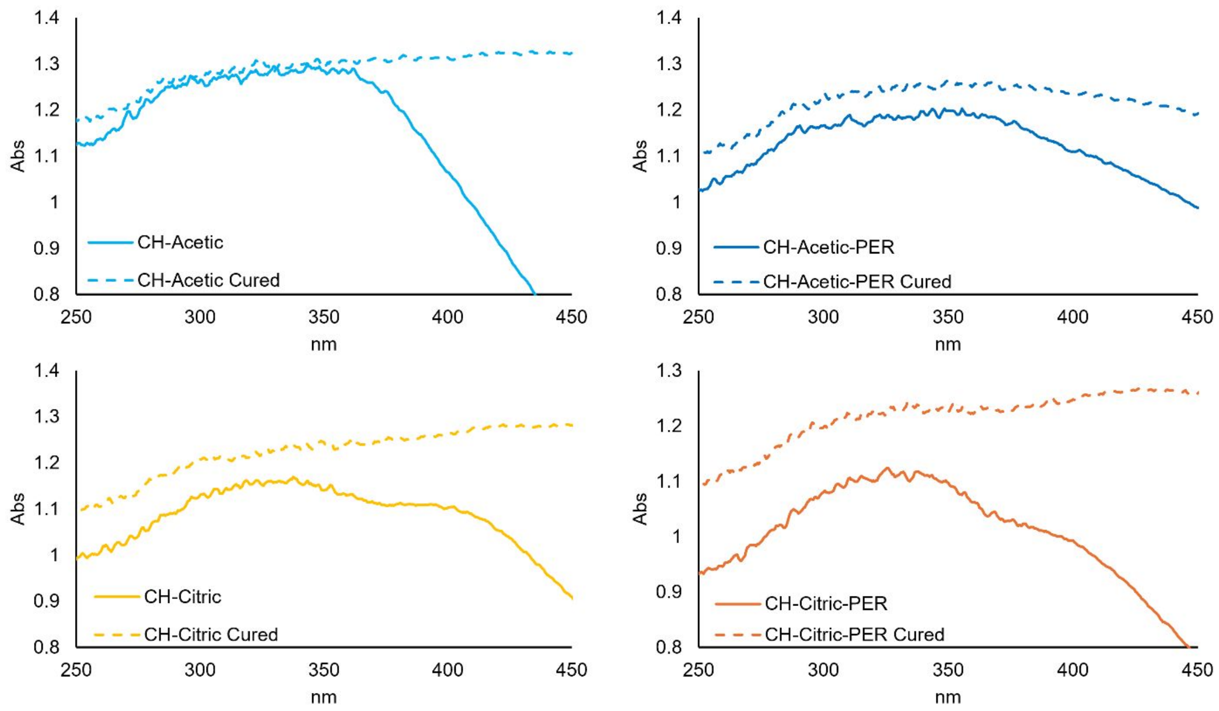

Figure S2. UV-Vis absorption spectra of each dried adhesive film.

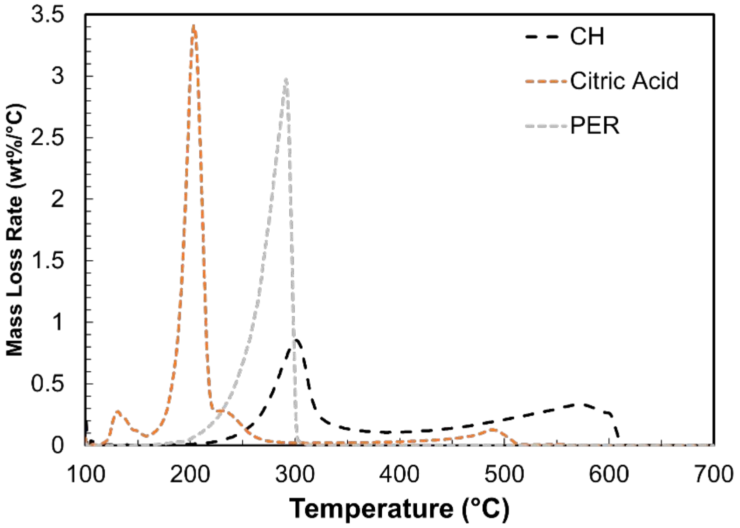

Figure S3. Differential thermogram of reagents used.

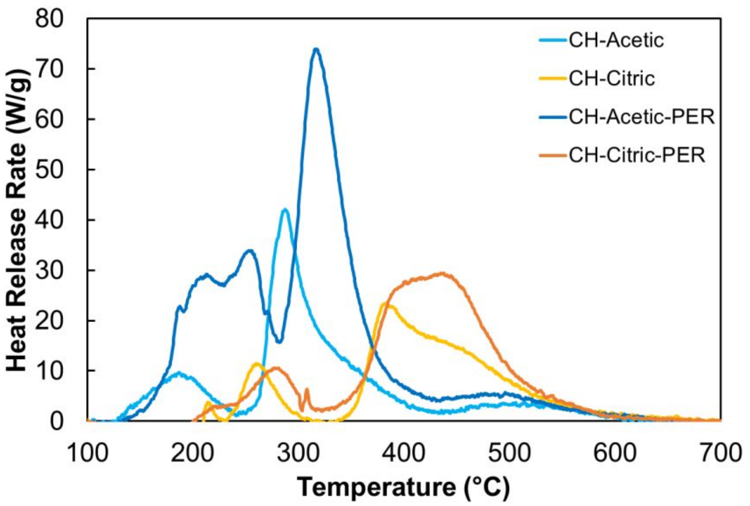

Figure S4. Microscale combustion calorimetry curves of each dried adhesive film.
